# Mesothelioma

**DOI:** 10.3390/cancers13133127

**Published:** 2021-06-23

**Authors:** Marcello Deraco

**Affiliations:** Director Peritoneal Surface Malignancies Unit, Department of Surgery, Fondazione IRCCS Istituto Nazionale Dei Tumori, Via Giacomo Venezian 1, 20133 Milano, Italy; marcello.deraco@istitutotumori.mi.it

Malignant mesothelioma is a disease affecting serosal surfaces derived from the mesothelium comprising the pleura, peritoneum, pericardium, and tunica vaginalis testis. Peritoneal mesothelioma (PM) accounts for 7–30% of all cases [1]. The incidence of malignant mesothelioma varies widely geographically. The highest rates are reported in the UK, Australia, and New Zealand, while some of the lowest reported rates are from Japan, Slovenia, and other countries in central Europe. The United States has an incidence in the middle range of about 1.94 and 0.41 per 100,000 for men and women, respectively [2,3]. There will be approximately 15,000 cases of PM diagnosed between 2005 and 2050 in the US [1] and 300–400 new cases of peritoneal mesothelioma are estimated to be diagnosed annually.

The most common diffuse malignant form of PM (DMPM) is characterized macroscopically by thousands of whitish tumor nodules of variable size and consistency that may coalesce to form plaques or masses, or layer out uniformly over the entire peritoneal surface. Although there might be an association of asbestos exposure with DMPM, this disease’s pathogenesis is mostly unknown. Patients usually present with advanced disease that causes abdominal pain or distension. As the disease progresses, patients die due to intestinal obstruction or terminal starvation within a year. In most patients, DMPM remains localized within the abdominopelvic cavity throughout its course.

Two decades ago, encouraging data on a multimodal approach that combines cytoreductive surgery (CRS) and hyperthermic intraperitoneal chemotherapy (HIPEC) were reported. According to a recent International Consensus Statement, CRS and HIPEC is currently regarded as the gold-standard initial treatment in selected patients with malignant DMPM [4] as it extends overall survival from a median of 9–13 months in patients undergoing palliative systemic chemotherapy [5,6,7] to 34–92 months [8,9,10,11].

This monograph’s objective is to present a comprehensive overview of the state-of-the-art data regarding the epidemiology, diagnostics, therapeutics, and pathophysiology of PM.

The current project brought together the efforts of the most prominent experts in peritoneal surface oncology and has the mission to provide clinicians and researchers worldwide with a piece of updated information that will serve to further optimize therapeutics, improve the prognosis, and deepen our knowledge of the disease’s biology in the years to come.
